# Views and experiences of ethnic minority diabetes patients on dietetic care in the Netherlands – a qualitative study

**DOI:** 10.1093/eurpub/cky186

**Published:** 2018-09-11

**Authors:** Mirjam J Jager, Rob van der Sande, Marie-Louise Essink-Bot, Maria E T C van den Muijsenbergh

**Affiliations:** 1 Nutrition and Dietetics, HAN University of Applied Sciences, Nijmegen, The Netherlands; 2 Department of Public Health, Academic Medical Centre, Amsterdam, The Netherlands; 3 Primary and Community Care, HAN University of Applied Sciences, Nijmegen, The Netherlands; 4 Department of Primary and Community Care, Radboud University Medical Centre, Nijmegen, The Netherlands; 5 Prevention and care programme, Pharos, National Centre of Expertise on Health Disparities, Utrecht, The Netherlands

## Abstract

**Background:**

Diabetes type 2 is more prevalent in people from ethnic minorities in the Netherlands, and outcomes of care are worse compared with other Dutch people. Dieticians experience difficulties in managing these groups in self-management and adherence to dietary advice. The aim of this study was to explore the views regarding a healthy diet and dietetic care among ethnic minority type 2 diabetes patients.

**Methods:**

Semi-structured interviews were held with 12 migrants with diabetes from Turkey, Morocco, Iraq and Curacao, who visited a dietician. Inclusion went on until saturation was reached. The interview guide was based on the Attitudes, Social influence and self-Efficacy (ASE) model and Kleinman’s explanatory model of illness. Interviews were held in the language preferred by the respondent. Transcripts were coded and thematically analyzed.

**Results:**

Several respondents expected a more rigorous, directive and technical approach of the dietician. All respondents acknowledged the importance of a healthy diet. What they considered healthy was determined by culturally influenced ideas about health benefits of specific foods. Important hindrances for dietary change were lack of self-efficacy and social support. Social influences were experienced both as supportive and a hindrance.

**Conclusions:**

Migrant diabetic patients’ opinions about healthy food are determined by culturally influenced ideas rather than by dietary guidelines. Dutch dietary care is not tailored to the needs of these patients and should take into account migrants’ expectations, cultural differences in dietary habits and specifically address the role of family.

## Introduction

Migration and belonging to an ethnic minority is a risk factor for developing type 2 diabetes (T2D).^1^ Ethnic minorities have a two to five times higher prevalence of T2D than the host European populations.^2^ The reasons for this higher prevalence are thought to be a complex interplay of genetic factors (e.g. in groups of South-East Asian descent) and environmental factors related to chronic stress and lifestyle; the latter explains why migrants suffer from diabetes more often than inhabitants of their country of birth.^3^^,^^4^ Eleven percent of the Dutch population consists of migrants, of whom the three largest groups originate from Turkey, Morocco and Surinam.^5^ Diabetes care is less effective and of lower quality in migrants and ethnic minorities than in other patients, leading to higher rates of complications and higher health care costs.^1^^,^^6^

In the Netherlands, healthcare for patients with T2D is concentrated in primary care following nation-wide accepted multi-disciplinary guidelines.^7^ These guidelines recommend dietary advice and counseling by a dietician. The guidelines promote the consumption of fruits, vegetables, wholegrain products and fish, and discourage the consumption of saturated fats and sugar-containing beverages.^8^ However, in practice the dietary advice is based on the Dutch food consumption patterns, and no guidance exists how to tailor dietary advices to the needs of migrant patients with different food culture, nor to their health literacy level. Half of the ethnic minority elderly have lower educational level, low mastery of the Dutch language and low health literacy.^9^

Healthcare professionals often experience difficulties in promoting effective self-management and adherence to dietary advice in migrant patients with diabetes^10^^,^^11^ due to language differences and low health literacy.^12^

Although it is generally accepted that culture has a strong influence on diet,^13^^,^^14^ little is known about how ethnic minority patients with diabetes think about dietetic care and a healthy diet. As these factors may also contribute to the communication problems, insight in patients’ views is crucial for the development of effective dietetic care, tailored to the patients’ needs.^15^ This study explores experiences and views of ethnic minority T2D patients regarding a healthy diet and dietetic care in order to generate information that may be used for the development of training for dieticians in culturally competent dietetic care.

## Methods

### Design

We adopted a qualitative research approach^16^ using in-depth semi-structured interviews.

The study was approved by the Medical Ethical Committee of the Central Committee on Research involving Human Subjects region Arnhem and Nijmegen (reference 2014/210).

### Study population

By purposive sampling, first generation migrant respondents from African, Asian or Latin American origin were recruited from nine Dutch dietetic practices in areas with a high proportion of migrant residents, aiming at maximum variation among participants regarding gender, age, ethnic background, level of education and proficiency of the Dutch language. Dieticians as well as researchers informed migrant patients in these practices orally and by information leaflets in different languages about the study and invited them to participate. It was explained that the interviews would be held in their preferred language.

After signing informed consent, the interview was planned. Recruitment ceased when theoretical data saturation was reached.^17^ Participants received a gift voucher valued at €20.

### Topic list

The topic list was based on a theoretical framework consisting of two models: the Attitudes, Social influence and self-Efficacy (ASE) model for explaining motivational and behavioral change,^18^ linking attitude, social norm, self-efficacy, knowledge, and barriers to health related behavior change, and the Explanatory model of illness by Arthur Kleinman^19^ that addresses the social and cultural influences on illness and health. In this study, we operationalized ‘cultural influences’ as the utterings of participants on values or habits which contributed to their cultural or religious background, or to their country of origin.

The topic list contained questions about knowledge of, opinions about and experiences of the participants and their families with diabetes, healthy food, dietary change and dietetic care.

The topic guide was tested by a Turkish dietician and bilingual Turkish and Moroccan researchers to ensure appropriateness of language used.

### Data collection

Six bilingual interviewers who spoke either Arab, Berber or Turkish were recruited and trained for the interviews. All interviews took place at participants’ home, to ensure a safe environment to talk freely. In several cases, children or spouses were also present.

All interviews were recorded, transcribed and translated by the interviewers.

### Data processing and analysis

The data were anonymized and processed by the computer software program Atlas.ti. The interviews were read twice to gain an overall impression of the material. The data were subjected to a deductive qualitative analysis using an iteratively developed coding frame based on the ASE model^18^ and the Kleinman explanatory model of illness.^19^ This framework was expanded iteratively with newly emerging codes. We report data in this paper following overarching themes; to give insight into the linkage with the models used, we mention the corresponding model between brackets. To ensure reliability, the first six interview transcripts were independently coded and analyzed by two researchers (M.J., M.v.d.M. or R.v.d.S.). Differences were discussed until concordance was reached.

## Results

Theoretical data saturation was reached after inclusion of 12 patients, differing in country of origin, language, age and educational level (see table 1). Eight interviews were held in the patients’ mother tongue, four in Dutch.

**Table 1:** Patient characteristics

Respondent	Country of origin	Sex	Age (years)	Length of stay in the Netherlands (years)	Education	Language during interview
R01	Turkey	F	54	33	Primary school	Turkish
R02	Turkey	F	59	35	Primary school	Turkish
R03	Iraq	F	51	22	Unknown	Arab
R04	Iraq	M	69	25	Secondary school	Dutch
R05	Turkey	F	51	28	Secondary school	Turkish
R06	Turkey	M	52	26	Primary school	Turkish
R07	Turkey	F	52	28	Secondary school	Turkish
R08	Turkey	M	unknown	36	Higher education	Turkish
R09	Iraq	M	44	10	Higher education	Dutch
R10	Curacao, Netherlands Antilles	F	54	15	Secondary school	Dutch
R11	Morocco	F	69	46	Primary school	Dutch
R12	Morocco	F	87	34	None	Berber

### Culturally influenced opinions about healthy food (Kleinman/ASE)

Most respondents had a general knowledge about foods to avoid or restrict in case of diabetes like carbohydrate rich products or fats. All respondents, including those who lived in the Netherlands for many years, were used to eating according to their cultural tradition. This was reflected in their opinions on healthy food. For instance, several respondents believed that bulgur was better than rice for diabetes patients. One woman explained that cooking rice in plenty of water was better, she believed the ‘bad’ compounds and sugar were drained off.

Furthermore, respondents mentioned examples of food with supposed beneficial effects on diabetes that reflected their cultural background, like kefir, water with lemon juice, lime juice with garlic, spicy salad leaves and okra.

R09: ‘I’ve said it to people. People, stop with the sugar! I’ve found a medicine, garlic with green lemon. You cut it all, make juice, cook it and drink it. Your glucose levels go down.’

One respondent explained he always had a bowl of dried fruits and nuts on his table (which is common in Arab countries), because of the beneficial effect of the unsaturated fats on his cholesterol level. He had difficulty losing weight, and seemed unaware of the fact nuts and dried fruits might increase his energy intake.

### Culture of hospitality and emphasis on family impede dietary changes (Kleinman/ASE)

Although most respondents knew they should change their diet and do more physical exercise, many of them found this difficult. The culturally determined importance of hospitality and of the role of the family were important barriers.

Nearly all participants stressed that eating, and eating until they are satisfied, were very important for them as they were used to do so in their culture.

R05: ‘If I can’t eat and have a full stomach, what is the difference between life and death?’
R07 and R10: ‘Eating is living’
R06: ‘But you know the flavour of sütlaç (rice pudding) and you love it, you have it in your genes.’

All respondents ate together with their families, as they were used to in their country of origin. Even when children had their own families, they frequently joined their parents for dinner. Family could be either a great support, or a hindrance in changing their lifestyle.

Sometimes, the whole family ate according to the diet guidelines of the diabetes patient.

R04: ‘Wife: The whole family adjusts to his diabetes. Respondent: uhuh, we all eat the same.’

Another woman explained how her family was a motivation for making healthier choices:

R05: ‘I felt guilty because I set a bad example for my children. Apparently pastries cause obesity. That’s why I quit making those.’

However, in many patients, this social norm of eating with family and friends was a hindrance for adherence to their diet. Some felt that they could not impose their dietary changes upon their families and then found it difficult to adhere to them.

R05: ‘My eldest son is very picky. I can’t starve them. (….) I say: as long as the children eat. I don’t find myself that important.’

One respondent explained how his new eating regime formed a barrier to visit his family in law. His wife often visited them alone:

R09: ‘Going alone is a bit strange. But I want to eat healthy. I stay at home. I eat salmon, cook myself and eat.’

Hospitality is a very important social concept for all respondents, which they linked to their culture. Almost all people described social situations which were accompanied by (an abundance of) food. They might be pressured to eat, as refusing food may be found unacceptable in some cultures.

R09: ‘Maybe they will talk badly. (….) We are afraid of gossip.’

The majority of respondents had difficulties to adhere to their diet when they went to their country of origin for holidays, which they frequently did. Family visits were an important part of the holiday, often accompanied by an abundance of food.

R01: ‘In Turkey I should also pay attention. But there you are more dependent on others, because you stay with other people. You eat whatever is cooked by them. That’s why I pay less attention.’

At the same time, respondents explained how it is culturally accepted to refuse foods when a person is ill.

R07: ‘But if you refuse something because of your illness than that person should respect that. Why would they say anything? If you are offered something and say very crudely “No, I really won’t eat that!”, that would be bad, but if I say “girl, I can’t eat this because of this and this illness”. They just have to respect that.’

### Lack of motivation due to low confidence in the dietician or in ones’ self (ASE)

Other important barriers to dietary change were a lack of confidence in the dietician’s advices and a lack of self-efficacy.

A few respondents had a rather negative attitude towards changing their diet, as they did not believe the advice of their dietician was worthwhile.

R07: ‘Ok, the truth is that I didn’t read it. Yeah my children read it but I just don’t listen. (…) No, it doesn’t interest me. (…) If you do it the way you should, you will die of hunger. I don’t agree with what we discussed at the dietician last time. I can’t and won’t do that and such a diet doesn’t exist.’
R06: ‘If I eat two slices of cheese once or twice a week, what does it matter if it’s 30 or 48 (percent fat) ? I don’t think that will do much for me.’

There were other respondents with a particular low belief in their own ability to adhere to their diet.

R03: ‘Interviewer: Do you ever refuse food because of your diabetes? Respondent: when they serve something I don’t like, I don’t eat it. Interviewer: And when it’s something you really like? Respondent: “I can’t refuse that. Then I eat. Like desserts”.’

### Adherence to a diet during Ramadan (Kleinman/ASE)

Believing Muslims with diabetes may be exempt from fasting during Ramadan according to the Surah al-Baqarah, verse 184-185. However, several Muslim respondents said they participated in fasting and tried to incorporate their diet guidelines into the meals they ate after sundown and adjusted their medication.

R03: ‘You know how during Ramadan a nice dessert is eaten after dinner. Now I hardly eat something sweet. After getting diabetes, I couldn’t do that anymore.’
R05: ‘Because you don’t eat in the afternoon, you don’t take your medication.’

The Muslims who did not participate in the fasting explained they did so because of low blood sugars, or because people with diabetes in general are exempted from fasting.

A few people spontaneously mentioned that they lied to their doctor about participating in Ramadan.

R05: I’m not allowed by my doctor, but I did it anyway. That’s why I lied. He asked me and I said no. He said you shouldn’t fast and I said ok. But I didn’t want that, I wanted to fast.’

### Dietary care does not always meet the expectations of migrant patients (Kleinman)

Most respondents respected their dietician and valued the advice they were given.

However, quite a few participants were very dissatisfied with the care that was provided by their dietician. The main criticism was that the advice was not strict enough nor based on the outcomes of medical tests.

R02: ‘She doesn’t say eat this and don’t eat that. (….) I said to the doctor, you sent me to the dietician, but she doesn’t do anything. She just weighs me, but she doesn’t do anything else.’

An advice that was based on mere conversation was not appreciated and trusted.

R06: ‘To understand me, you have to do some physical tests, right? You have to look at my blood work (….) If you give me a diet list by just talking to me, then I don’t think it’s very useful to see you anymore.’
R07: ‘This dietician started with questions right away, without doing any measurements. That’s why my interest for the conversation was minimal.’

### Language is not a problem

Almost all patients felt fairly confident that they understood what the dietician said and asked. However, during the interviews signs were noted that there was a language barrier. Some patients said they had difficulty with answering questions from the dietician, but they did not indicate this as a problem. One respondent mentioned that she found it difficult to explain the specific Turkish dishes she ate.

## Discussion

To our knowledge this is the first study that explored opinions and experiences of migrant diabetes patients with dietetic care and dietetic advise.

All 12 respondents knew they should change their diet, but found it very difficult to do so. Their opinions about healthy food were heavily influenced by their culturally determined eating habits. Specific challenges mentioned were culturally bound obligations of hospitality that involved an abundance of food, and Ramadan for Muslims. Sometimes motivation was lacking due to lack of confidence in the dietician or as a result of low self-efficacy. Dietetic care did not meet the expectations of a substantial part of the respondents. They would prefer a more technical medical approach, based on tests.

This study contributes to the evidence that dieticians should be attentive to and ask about specific cultural beliefs and eating patterns of migrant patients.

Dieticians need to know what drives a person to eat the way they eat, in order to make sustainable changes to their diet. The ASE model explains behavior through the determinants attitude (beliefs), social influence and self-efficacy.^18^ Our results show cultural background is important regarding beliefs about healthy food and social influence. This is in line with previous research among Surinamese diabetes patients in the Netherlands who found it difficult to reconcile Dutch dietary guidelines with Surinamese cooking and eating patterns.^20^ Furthermore, elaborate cooking and serving various snacks have an important role in hospitality and people may find it impolite to refuse the foods offered.^15^ The conflict of hospitality and adhering to healthy diet was also found in a study among Turkish and Moroccan participants.^13^ However, our respondents emphasized they could refrain from hospitality obligations because of their diabetes. Social networks contribute to diabetes management in providing practical and emotional support.^21^^,^^22^

This will be ever so much the case in patients from more collectivistic cultures in which the role of the family is even more important.^23^ However, social networks can also be felt as a burden. This implicates that dieticians should consider and discuss with the patient the involvement of family members in the diet counseling process.

Culture also influences patients’ expectations of healthcare and healthcare providers.^20^ Kleinman describes how eliciting the patient’s expectations and preferences is an important tool for facilitating cross-cultural communication, ensuring patient understanding, and identifying areas that need to be negotiated.^19^ A key issue in our respondents was that they expected a more technical medical approach to dietetic care. This finding is in line with other studies on the healthcare preferences of migrants in the Netherlands that emphasize the importance of a physical examination in medical encounters,^24–26^ and reflects the medical culture in their country of origin.^27^ In Turkey for instance, dieticians work primarily in a hospital setting and not in a primary health care facilities.^28^ To build the necessary relation of confidence and trust, healthcare professionals need to address the expectations and preferences of their patients.^24^

Culturally sensitive health education may positively influence lifestyle in ethnic minority patients.^29^^,^^30^ This requires the involved healthcare providers to be culturally competent, which is generally defined as possessing the combination of knowledge, attitudes and skills necessary for delivering high-quality care to an ethnically diverse patient population.^31^^,^^32^

Unexpectedly, and contrary to many other studies,^12^ the respondents did not mention language problems as a barrier in their contacts with their dietician. It might be possible that they were not aware of this barrier, as it is known that non-native speaking patients often overestimate their language proficiency.^33^ So, it remains important for dieticians to assess the language skills of their patients and involve interpreter services when needed.^34^

### Strengths and limitations of this study

This is the first study that evaluates the views and experiences of migrants in the Netherlands on care by a dietician. Most literature about patients’ views on and experiences with diabetes care focus on doctors and nurses.

Another strength is the fact we employed bilingual, trained interviewers. This facilitated the inclusion of first generation ethnic minority patients; a group generally hard to reach in research due to language barriers, illiteracy, and mistrust of research.^35^

Since we recruited patients who visited their dietician, we miss the views of ethnic minority diabetes patients that are not referred to a dietician, or did not follow this referral. It seems unlikely that these patients face less barriers in adherence to healthy diet, but it is possible they experience additional barriers in adherence, for instance due to a language barrier.

We deliberately included patients with a broad range of ethnicities, as we were not looking for culture-specific elements and want to avoid stereotyping. We believe any contact between a dietician and a patient can be regarded as cross-cultural, as nearly always there are differences in social and educational backgrounds. However, the importance of knowledge about general aspects of cultural background, and skills to discuss these with the patient becomes greater when cultural distance between a healthcare provider and patient are larger. A person-centered approach, with specific attention to ethnic and cultural aspects is called culturally competent care.^32^ Cultural competences at the provider level are defined as the knowledge, attitudes and skills necessary to provide good quality of care for ethnic minority patients.^32^ This approach has the potential to reduce disparities in health care by seeking to equalize power between providers and patients.^32^^,^^36^ Person-centeredness and cultural competence are highly congruent at the healthcare provider level.^37^ The potential complexity of communication with patients from an ethnic minority group – due to language barriers, cultural distance or influence of stereotypes – asks for distinct qualities, or a ‘PLUS’ in the generic person-centered approach.^32^^,^^36^^,^^38^ For example, dieticians need to ask about participation in Ramadan, a question that might not arise when treating a non-Muslim patient. Knowledge about food culture and recipes is necessary to know what probing questions to ask when a dietician assesses food intake. Furthermore, dieticians must be aware of views among migrants about the role of the dietician and be able to adapt their approach accordingly. Training can improve the cultural competences of health care providers.^39^

More research is needed to elicit the views of dieticians on the care for migrant diabetes patients. Furthermore, observations of consultations could provide information on the actual interaction between dieticians and migrant diabetes patients.

## Conclusions

Migrant diabetic patients’ opinions about healthy food are determined by culturally influenced ideas rather than by dietary guidelines. Dutch dietary care is not tailored to the needs of these patients and should take into account migrants’ expectations, cultural differences in dietary habits and specifically address the role of family.

## Funding

This work was supported by the Netherlands Organisation for Scientific Research (NWO), grant number 023.003.104.

## Disclaimer

The authors confirm all patient/personal identifiers have been removed or disguised so the patient/person(s) described are not identifiable and cannot be identified through the details of the story.

*Conflicts of interest*: None declared.

**Key points**

- Patients in ethnic minority groups expect a more rigorous, directive and technical approach to dietetic care
- Patients in ethnic minority groups have culturally influenced ideas about healthy foods for diabetes
- Social support, hospitality and self-efficacy are an important influence on maintaining a healthy diet amongst these patients
- Training of dieticians should focus on eliciting the patients’ expectations towards dietetic care and involving family in the treatment.
